# Understanding the effect of sociodemographic and psychological latent characteristics on flex-route transit acceptance

**DOI:** 10.1371/journal.pone.0279058

**Published:** 2023-02-06

**Authors:** Jingcai Yu, Wenquan Li, Jin Zhang, Rongrong Guo, Yan Zheng

**Affiliations:** School of Transportation, Southeast University, Nanjing, China; Central South University, CHINA

## Abstract

Flex-route transit (FRT) has significant advantages in low-demand areas. Existing studies have focused on practical experience, strategic planning, and operational planning. Few studies have addressed the effect of sociodemographic and psychological latent characteristics on the acceptance of FRT. This study aims at exploring the effect of sociodemographic and psychological latent characteristics on FRT acceptance. To finish the goal, a household survey is conducted from April to May 2020 in Nanjing, China. The survey includes sociodemographic characteristics and observed variables of individuals. Firstly, the study extracts six psychological latent characteristics to reflect individuals’ attitudes based on previous and mature researches in the field of technology acceptance model (TAM) and theory of planned behavior (TPB). Then, a multiple indicators and multiple causes (MIMIC) is applied to calculate six psychological latent characteristics. Finally, an integrated model, consisting of the MIMIC and a binary logit model (BLM), is applied to match sociodemographic and psychological latent characteristics. The BLM with sociodemographic characteristics is developed as the reference model to compare the effects of psychological latent characteristics. Results show that psychological latent factors play a significant role in estimating the effect on FRT acceptance. Results of the integrated model show that the parameter of car is -0.325, displaying individuals with private cars are more reluctant to use FRT. Therefore, restricting private cars is an effective measure to facilitate FRT. Improving flexibility (0.241) is a significant measure to facilitate FRT. Findings are expected to facilitate decision-making of transport planners and engineers, and therefore enhance the service of the FRT system.

## 1. Introduction

Public transit is an effective option for releasing traffic congestion and reducing vehicle emissions [1–3]. The traditional fixed-route transit has been successfully operated in many cities. The advantages of such a mode might be subjected to the low-demand areas. For instance, the fixed schedule and routes could increase the departure interval of buses in low-demand areas, and in turn affect the comfort of passengers [4, 5]. In this context, flex-route transit (FRT) is regarded as a promising public transit mode [5, 6]. The operation data of the FRT in Merrill indicate that FRT has a positive impact on the transport system, especially in low-demand areas [7]. The ridership in Virginia is found significantly increase (about 354%) after operating the FRT system, and about 82 percent of passengers are satisfied with the service of FRT [6].

FRT can offer services with a base route, which has several compulsory stations (i.e., checkpoints). The bus has to stop at the compulsory station on time. Thus, FRT not only can punctually offer services at the checkpoints, but also can serve the passengers at not-checkpoints (i.e., request) using the slack time [8]. Compared with traditional fixed-route transit, FRT is more flexible because it allows the bus to deviate from the base route and provide door-to-door services [9]. Also, such a mode is more economical when offering door-to-door services due to the high capacity compared with dial-a-ride systems or taxis [10].

Previous studies mainly focused on three aspects: practical experience, strategic planning, and operational planning [11–13]. For practical experience, FRT was applied primitively during the 1970s. Then it was implemented in Merrill, Wisconsin [7]. The FRT can offer a feasible traffic mode in lower-density travel areas and serve passengers more effectively. Besides, FRT was applied successfully in OmniLink, Prince William County, Virginia [14]. The ridership of public transit in these areas has been increasing steadily since the introduction of the FRT. Moreover, the efficiency of the FRT improved significantly with the help of ITS technologies. For example, vehicle location can be tracked in real-time and information can be shared by interaction platforms between drivers and dispatchers by ITS technologies [15]. For strategy planning, which aims to apply an appropriate transit system in the different service areas and actual demand levels. Therefore, a decision method between traditional fixed-route and FRT in various demand densities was presented [4, 16]. Furthermore, a mixed transit system incorporating the advantages of fixed-route transit and FRT was proposed. Results indicated that such a mixed system is superior to other systems [17]. Several studies have been conducted on the scheduling planning of FRT [11, 12, 18, 19]. For instance, a real-time operation route of the bus was designed based on the route design and service levels of FRT [8]. The time schedules should be consistent with predetermined timetables before every departure. Several linear integer formulations, including three various planning policies, are proposed to solve the scheduling planning [17]. In addition, an insertion algorithm is presented to address the static and dynamic scheduling of the operation of FRT [18, 19].

Several studies have demonstrated that the operation and planning of a new travel mode are significantly related to the individuals’ acceptance of it [20, 21]. Indeed, several researchers believe that acceptance of a new travel mode is related to sociodemographic characteristics, such as gender, age, income, and education [22, 23]. In addition, travel behavior is also relevant to psychological factors, such as comfort, use willingness, and perceived barriers [24, 25]. FRT acceptance is related to influencing factors due to the features of public transit. However, few studies identify the effect of influencing factors on FRT acceptance. Therefore, it is worth exploring the effect of sociodemographic characteristics and psychological latent factors for developing FRT.

To finish this goal, the study conducted a household survey to collect sociodemographic characteristics and observed variables. Based on previous and mature researches in the field of technology acceptance model (TAM) and theory of planned behavior (TPB), the study extracts six psychological latent factors (i.e., comfort, flexibility, perceived barriers, personal barriers, subjective evaluation, and use willingness). Such latent factors can be calculated by a multiple indicators and multiple causes (MIMIC). The integrated model, including the MIMIC and a binary logit model (BLM), is used to address the effect of sociodemographic and six psychological latent characteristics. The contribution of the study is to identify the effects of socioeconomic and psychological latent factors on FRT acceptance by applying an integrated model, and further make some policy recommendations to facilitate FRT.

The rest of the paper is organized as follows. The survey data are described in Section 2. Section 3 focuses on the research methodology. The results and discussions are conducted in Section 4. Section 5 concludes the contributions and directions for further research.

## 2. Survey data

The household survey was conducted in the fringe of the city of Nanjing (China) to collect sociodemographic and observed variables of respondents in low-demand areas on FRT. Nanjing is located in southeast China, with a population of 9.31 million and an area of 6540.53 km^2^ in 2020 [26]. The data originated from the survey from April to May 2020 by the School of Transportation, Southeast University.

1290 questionnaires were randomly distributed to respondents. To improve the quality of the survey, the questionnaire did not include respondents’ private information (e.g., name and ID card). The survey explained that the aim of the survey was to develop FRT. Respondents answers were helpful in conducting the study. Respondents filled in the questionnaire after they understood the survey. The final survey referred to two main points: (1) Sociodemographic characteristics of respondents; (2) Observed variables of respondents. The study selected six psychological latent factors (i.e., comfort, flexibility, perceived barriers, personal barriers, subjective evaluation, and use willingness) based on previous and mature researches in the field of TAM and TPB [10, 27, 28]. 18 observed variables were defined to reflect psychological latent factors on FRT by the Cattell’s Scree Plot method [25, 29].

972 valid samples were obtained after excluding invalid data (the effective rate was 75.35%). Table 1 describes sociodemographic characteristics and familiarity with FRT of respondents. The respondents were randomly selected during the survey and answered the point of disapproval or approval for each question using the Likert seven-scale method (1–7), using “completely disagree”, “partly disagree”, “disagree”, “neutral”, “agree”, “partly agree” and “completely agree” were used to rate the attitudes. Table 2 shows the description of 18 observed variables with the Likert seven-scale method [30].

**Table 1:** Descriptive statistics: Sociodemographic characteristics of respondents.

Sociodemographic characteristics	Valid sample (N = 972)
	Distribution (%)
Age [years] (12~18, 19~30, 30~40, 40~50, 50~60, 60+)	(10.9, 18.1, 25.4, 19.8, 17.1, 8.7)
Gender (female, male)	(46.6, 53.4)
Education [years] (9-, 10~12, 13+)	[22.5, 47.8, 29.7)
Individual income [¥/month] (5000-, 5001~7000, 7001~9000, 9001+)	(20.4, 36.7, 24.3, 18.6)
Child (12 years or younger) (0, 1+)	(36.4, 63.6)
Car availability (0, 1+)	(60.7,39.3)
Familiarity (1, 2, 3, 4, 5, 6, 7)	(27.2, 24.3, 23.6, 10.2, 7.4, 4.2, 3.1)

**Table 2:** The description of observed variables.

Observed variables		Distribution (%)							Likert scale	
		1	2	3	4	5	6	7	Mean	SD
CO1	I can obtain a seat while taking.	1.7	4.3	2.4	30.3	32.6	19.1	9.6	4.83	1.05
CO2	I can do something while taking.	3.5	4.7	14.9	11.8	27.5	26.1	11.6	4.80	0.93
CO3	Traveling with FRT is easy.	3.9	5.7	11.3	40.4	23.4	13.1	2.3	4.22	1.52
FL1	The accessibility of using FRT is higher.	4.3	2.2	6.3	56.3	16.2	12.1	2.7	4.25	1.28
FL2	The convenience of FRT is good.	10.0	7.9	6.9	37.4	19.4	10.0	8.3	4.12	1.74
FL3	The wait time for FRT is short.	1.8	4.3	5.2	33.0	18.9	20.3	16.5	4.90	1.47
PB1	FRT does not exist when I want to ride.	4.8	10.5	7.4	32.6	20.8	13.7	10.3	4.36	1.65
PB2	FRT does not go to my location for service.	1.5	2.5	10.8	46.4	16.6	13.3	9.0	4.50	1.10
PB3	FRT is often late.	2.5	5.6	7.5	50.5	15.2	11.6	7.2	4.34	1.28
PE1	Economic conditions aren’t enough.	1.6	3.3	8.2	61.2	15.9	7.1	2.7	4.19	1.13
PE2	Travel environment isn’t suitable for FRT.	9.0	5.0	13.6	36.8	19.8	7.3	8.6	4.10	1.76
PE3	Physical condition isn’t suitable for FRT.	6.3	8.1	8.1	43.4	15.8	9.2	9.2	4.19	1.63
SE1	FRT has benefits for the environment.	10.6	9.5	8.2	37.2	20.9	7.9	5.7	3.95	1.39
SE2	Parking space isn’t necessary for consumers.	2.8	7.1	12.7	34.4	24.7	12.1	6.2	4.32	1.38
SE3	FRT can relieve traffic congestion.	5.9	5.8	11.8	42.7	19.2	13.5	1.1	4.08	1.32
UW1	Friends think that I should use FRT to travel.	5.6	10.5	9.5	39.5	19.0	13.7	2.3	4.06	1.91
UW2	I will encourage friends to use FRT.	9.6	3.5	9.0	38.1	23.8	11.8	4.2	4.15	1.21
UW3	The availability rate of FRT is high.	7.1	9.9	12.1	37.2	19.3	6.3	8.2	4.03	1.63

To testify the reliability and validity of the survey data, and Kaiser-Meyer-Olkin (KMO) sample measurements and Bartlett ball tests are used. KMO values are all higher than 0.5, the Bartlett ball test is less than 0.05, and Cronbach’s Alpha is higher than 0.7, which indicate the reliability and validity of the data are ideal [31–33]. Results are shown in Table 3.

**Table 3:** The reliability and validity test of the samples.

Psychological latent variable		Observed variables	KMO	Bartlett	Cronbach’s Alpha
Comfort	The comfort of respondents on FRT	CO1	0.724	0.001	0.784
		CO2			
		CO3			
Flexibility	The flexibility of respondents on FRT	FL1	0.641	0.000	0.817
		FL2			
		FL3			
Perceived barriers	The perceived barriers of respondents on FRT	PB1	0.614	0.002	0.712
		PB2			
		PB3			
Personal barriers	The personal barriers of respondents	PE1	0.657	0.001	0.828
		PE2			
		PE3			
Subjective evaluation	The subjective evaluation of respondents on FRT	SE1	0.612	0.000	0.753
		SE2			
		SE3			
Use willingness	The use willingness of respondents on FRT	UW1	0.550	0.000	0.759
		UW2			
		UW3			

As shown in Table 3, the minimum KMO and Cronbach’s Alpha are use willingness (0.550) and perceived barriers (0.712), respectively. The maximum Bartlett’s test was perceived barriers (0.002). It indicated that the applied data were statistically significant.

## 3. Methodology

**Fig 1:**
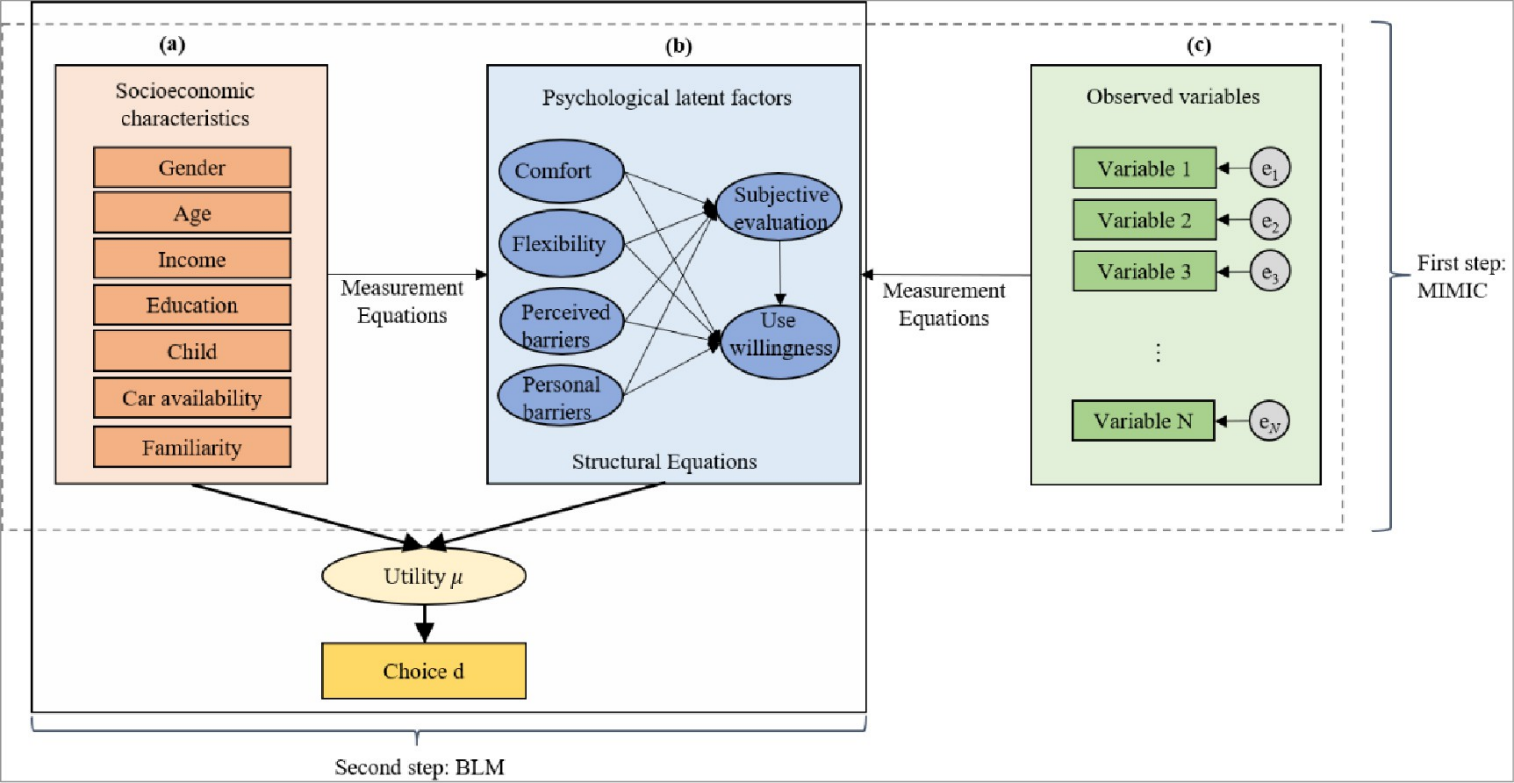
The framework of the integrated model.

To examine the effect of sociodemographic and psychological latent characteristics on FRT acceptance, an integrated model is applied. The integrated model consists of MIMIC and BLM [34, 35], shown in Fig 1. The MIMIC can explain the relationship between psychological latent factors and explanatory variables (i.e., observed variables and socioeconomic characteristics) [31, 32, 36]. The BLM describes the mode choice. The implementation of the integrated model can be divided into two steps: the first step is to estimate psychological latent factors (dashed box), and the second step is fulfilled to evaluate the discrete choice by the BLM (solid box).

The MIMIC model used in this study includes three types of variables: sociodemographic characteristics, observed variables, and psychological latent variables. Rectangular boxes in Fig 1(A) represent sociodemographic characteristics (i.e., gender, age, income, education, child, car availability, and familiarity). These factors are used to analyze the relationships with latent variables. It is a measurement equation. Ellipses boxes in Fig 1(B) represent psychological latent factors (i.e., comfort, flexibility, perceived barriers, personal barriers, subjective evaluation, and use willingness). It is the structural equation that is applied to analyze the relationship between latent variables. Rectangular boxes in Fig 1(C) are observed variables that can be obtained from the survey data. They are used to extract latent variables. The process is also a measurement equation.

Circular boxes represent the error term. The single-headed arrows represent the causal effects between variables, and all the path coefficients are estimated. The relationship of factors provides crucial information in understanding FRT acceptance. The schematic structure of the MIMIC model is shown in Fig 1.

The MIMIC can be expressed as follows:

y=Λη+ν (1)

η=Γx+ζ (2)

Where **η** is the vector of psychological latent factors; **y** is the observed indicator vector of **η**, such as CO1, CO2 and CO3; **x** is the vector of sociodemographic characteristics, such as age and education. **Λ** and **Γ** are the unknown parameter matrix between psychological latent factors and sociodemographic characteristics, and latent variables and observed variables, respectively. ν and ζ denote the measurement error, respectively (see S1 Appendix).

Usually, the utility *u*_*i*_ of traveler *i* is obtained as follows:

$$\mu_{i}=a_{i}x_{i}+b_{i}\eta_{i}+\varepsilon_{i}$$ (3)

$$d=\left\{ \begin{array}{l} 1,u=\max\left(\mu_{i}\right)\\ 0,other \end{array}\right.$$ (4)

Where **x**_***i***_ is the vector of sociodemographic characteristics; **η**_***i***_ is the vector of latent factors; **a**_***i***_ and **b**_***i***_ are vectors of unknown parameters to be evaluated, respectively; ε_*i*_ = (ε_1_,…,ε_*I*_) is measurement errors.

## 4. Results and discussions

### 4.1 Multiple indicators and multiple causes (MIMIC)

**Fig 2:**
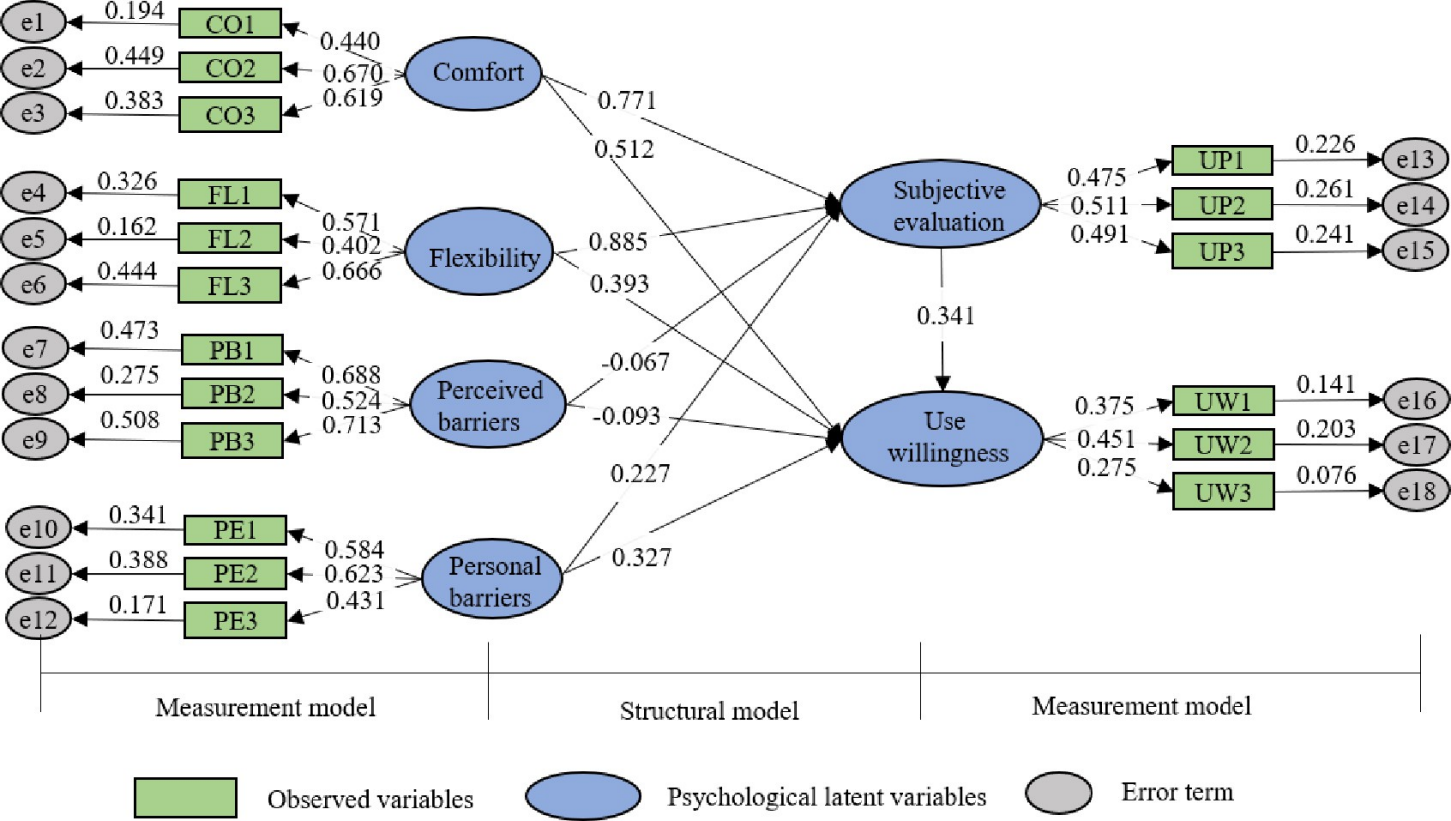
Estimation results between psychological latent variables and observed variables.

The MIMIC is applied to calculate psychological latent factors by the Analyze of Moment Structures (AMOS) software. The relationships between psychological latent factors and observed variables are shown in Fig 2. In Fig 2, the goodness of fit index (GFI) is 0.913, and the adjusted goodness of fit index (AGFI) is 0.926, which all exceeds the ideal value of 0.9. Meanwhile, the root means the square error of (RMSEA) is 0.028, less than 0.05. These indices manifest that MIMIC has a goodness-of-fit [35]. The structural model explores the relationship between psychological latent factors, such as the relationship between comfort and subjective evaluation. The measurement model shows the relationships between latent and observed variables (e.g., flexibility and FL1, FL2, FL3).

As shown in Fig 2, several psychological latent factors are significantly related to use willingness. The comfort and flexibility are positively related to subjective evaluation and use willingness, indicating that individuals may prefer to take FRT if the comfort (e.g., one seat for every passenger) and flexibility (e.g., door-to-door service) of FRT are facilitated. Similarly, personal barriers have a positive impact on use willingness, implying that FRT may be more popular among individuals with lower independent mobility (e.g., the handicapped and the elderly). Besides, perceived barriers have a negative impact on use willingness, meaning that individuals may not choose FRT if the service quality of FRT is lower (e.g., FRT can’t arrive at the location punctually).

The relationship between psychological latent factors and sociodemographic characteristics is explored, shown in Table 4. The *t* values are expressed in parentheses.

**Table 4:** The relationship sociodemographic characteristics and psychological latent factors.

	Comfort	Flexibility	Perceived barriers	Personal barriers	Subjective evaluation	Use willingness
Gender	–	–	–	–	0.006 (1.98)	0.017 (2.08)
Age	0.115 (1.99)	0.063 (4.21)	0.201 (4.39)	0.143 (5.72)	0.106 (3.21)	0.034 (2.69)
Income	-0.215 (-6.52)	-0.035 (-3.52)	–	–	-0.133 (-3.07)	-0.014 (-2.15)
Education	–	–	–	–	–	–
Children	-0.041 (-2.72)	-0.049 (3.85)	–	–	–	–
Car	-0.351 (-2.63)	-0.239 (-6.32)	–	–	-0.331 (-8.07)	-0.213 (-7.63)
Familiarity	–	–	-0.063 (-3.51)	–	0.019 (4.36)	0.042 (5.08)

*Note*: “-” expresses that the variable is not significant.

As shown in Table 4, The *t* values of latent variables are statistically significant at the 5% level. The findings imply that the estimation results are reasonable. Use willingness is related to gender, age, and familiarity with FRT, indicating that females, elderly individuals and individuals with more familiarity with FRT have a more tendency on FRT acceptance. This could be attributed to the difference in the perception on FRT.

### 4.2 Integrated model

The integrated model is applied to examine the effect of explanatory variables on FRT acceptance. BLM, as a reference, is used to compare the effect of psychological latent factors. SPSS is utilized to celebrate the parameters of the two models. The McFadden pseudo R^2^ is applied to estimate the goodness-of-fit of the model. Akaike information criterion (AIC) is utilized to compare the good-of-fit of the two models [37, 38]. For McFadden R^2^, the result is acceptable when the McFadden R^2^ of the model is more than 0.2 [38]. Variables that have statistically significant effects are saved by the stepwise model building process. The results of the BLM and integrated model are shown in Table 5.

**Table 5:** The evaluation results of BLM and integrated model.

Variable name		BLM		Integrated model	
		Estimate	*t*-statistics	Estimate	*t*-statistics
Exogenous variables	Gender	0.016	2.09	-	-
	Age	0.065	3.32	0.026	2.35
	Income	-0.113	-4.50	-0.045	-2.84
	Education	0.019	3.83	0.034	2.66
	Children	0.032	2.29	-	-
	Car	-0.451	-4.73	-0.325	-7.17
	Familiarity	0.075	3.65	0.098	3.56
Latent variables	Comfort	/	/	0.182	2.36
	Flexibility	/	/	0.241	4.74
	Perceived barriers	/	/	-0.142	-3.75
	Personal barriers	/	/	0.133	2.51
	Subjective evaluation	/	/	0.137	2.01
	Use willingness	/	/	0.121	2.06
*N*		972		972	
*p*		7		13	
*ln l*		-532.51		-451.6	
McFadden pseudo R^2^		0.261		0.368	
AIC		1.11		0.94	

*Note*: The Akaike information criterion (AIC) can be defined as follows: AIC=−(2/N)*lnl+2p/N. Where *p* is the number of parameters and *N* is the sample size.

As shown in Table 5, the McFadden pseudo R^2^ of the BLM and integrated model are 0.261 and 0.368, respectively. Both values are more than 0.2. Meanwhile, the AIC of the integrated model (0.94) is lower than that of BLM (1.11). Thus, the goodness-of-fit of the integrated model is better compared to BLM. Results indicate that psychological latent factors play a significant role in estimating the effects.

(1) The effect of sociodemographic characteristics

The acceptance of individuals on FRT is related to age, income, education, car availability and familiarity with FRT. Income (-0.045) and car availability (-0.325) are negative to FRT acceptance. The results indicate that the probability of FRT acceptance would be lower for individuals with higher income or private cars. The probability of FRT acceptance may be higher for lower-income individuals, which is reasonable due to the lack of the ability to purchase a car. The results are consistent with [39]. Individuals with private cars tend to use a private car due to the advantages of the short travel time compared to FRT. The findings are aligned with [40]. The FRT acceptance is positively related to age (0.026), education (0.034) and familiarity with FRT (0.098), and familiarity with FRT has the most positive impact. Respondents who have a higher familiarity with FRT, have a higher possibility of accepting FRT. The results may be logical that they have more understanding of the advantages of FRT. The results are consistent with [41]. Elderly individuals are inclined to accept FRT. This might be that short walking time, resulting from the advantage of door-to-door service, is crucial for them. The probability of accepting FRT would increase for individuals with higher education. The reason could be that FRT acceptance be higher for them. The findings are consistent with [41, 42]. However, no obvious evidence shows that gender and age have a significant effect on FRT acceptance in the integrated model. The reason could be that their effects are included in psychological latent factors, which are relevant to sociodemographic characteristics. The results for sociodemographic characteristics indicate that restricting private car ownership may be the most effective measure to improve the market share of FRT for sociodemographic characteristics.

(2) The effect of psychological latent factors

Six psychological latent factors are significantly related to FRT acceptance based on the results in Table 5. Perceived barriers (-0.142) have a negative influence on FRT acceptance, implying that the probability of accepting FRT may increase when perceived barriers decrease. For instance, perceived barriers may be decreased since the scheduling of FRT is strictly implemented to serve individuals punctually. The results are consistent with [42]. In addition, these psychological latent factors (i.e., comfort, flexibility, subjective evaluation, personal barriers, and use willingness) have a positive effect on FRT, and flexibility (0.241) is the most positive variable. Individuals have a stronger tendency to accept FRT when a higher comfort is available. The reason could be that comfort is regarded as a crucial variable for individuals. Enhancing the comfort of FRT is an effective measure to facilitate FRT acceptance, such as large-capacity buses are operated to enhance comfort by providing at least a seat for each. The findings are aligned with [43–45]. The positive relationship between flexibility and FRT acceptance indicates that increased flexibility of FRT could help motivate individuals’ demand. The flexibility of FRT can be enhanced by shortening the waiting time for individuals. The results are coinciding with [43, 45]. Individuals with higher personal barriers are more willing to accept FRT due to the advantage of short walking distance. For example, individuals with disabilities have a stronger tendency to accept FRT, since they can take FRT at any location rather than walk to the bus station. The findings are consistent with [39, 46, 47]. Subjective evaluation is positively related to FRT adoption. Thus, subjective evaluation should be considered an influential variable for developing FRT. In other words, individuals with higher subjective evaluation are more likely to accept FRT. For instance, FRT not only reduces private car ownership and alleviates traffic congestion due to the large-capacity attribute of public transit. The findings are consistent with [42]. Furthermore, use willingness has a positive effect on FRT acceptance. It could be attributed to the effects of their friends and relatives. The results are consistent with [43, 47].

## 5. Conclusions

This study aims at exploring the effect of sociodemographic and psychological latent factors on the acceptance of FRT. Sociodemographic characteristics are collected by a survey in Nanjing, 2020. Based on TAM and TPB, the study extracts six psychological latent factors, which are calculated by the MIMIC. The integrated model is employed to match sociodemographic and psychological latent factors, and further estimating their effects. The binary logit model (BLM) is a reference model to compare the effects of psychological latent factors. Differences between the two models do exist when consider psychological latent factors. The McFadden pseudo R^2^ of the integrated model is 0.368, which higher than the McFadden pseudo R^2^ of the BLM (0.261). In addition, The AIC of the integrated model is 0.94, which lower than the AIC of the BLM (1.11). The results confirm the superiority of developing model considering psychological latent factors.

For sociodemographic characteristics, results indicate that restricting private car ownership (-0.325) is the most effective measure to improve FRT acceptance. Policies (such as increasing vehicle purchase tax and congestion charging fee) can be implemented to reduce private car ownership to improve the FRT acceptance. Results also show that individuals who are familiar with FRT are more willing to finish their trips by using FRT. Also, age and education are found to be positively correlated to FRT acceptance. In contrast, high-income shows a negative influence on FRT acceptance. No evidence indicates that gender and age are related to FRT acceptance. For psychological factors, comfort, flexibility, subjective evaluation, personal barriers, and use willingness are significantly correlated to FRT acceptance. In particular, flexibility (0.241) is more sensitive. Therefore, measures can be made to enhance flexibility (such as reducing the waiting time of individuals by increasing the buses or shortening the departure intervals.), so that the FRT acceptance might be improved. Perceived barriers has a negative impact, which can be indicative of the policies and strategies that can improve the service of the FRT system. Also, measures that can enhance the promotion of FRT to the public are crucial.

The study sheds light on the effect of sociodemographic and psychological latent characteristics on the FRT acceptance by applying an integrated model. The study shows the necessity of identifying determinants that affect the acceptance of individuals on FRT. Some future research efforts are still needed. First, sociodemographic and psychological latent characteristics are selected based on other behavior research of the public (such as bicycle and carsharing systems), TAM and TPB [27, 28, 40, 41]. Factors such as the efficiency of FRT can be considered in future studies. Also, the general public usually takes time to recognize and adapt to new infrastructure [42, 45–48]. It is worth exploring the effect of the contributory factors on the stage transition of the FRT behaviors.

## Supporting information

S1 Data (XLSX)

S1 Appendix Variables and equations. (DOCX)
