# Supplementary material for: Understanding the effect of sociodemographic and psychological latent characteristics on flex-route transit acceptance
Source: PLoS One. 2023 Feb 6;18(2):e0279058. doi: 10.1371/journal.pone.0279058 (PMC9901740; doi:10.1371/journal.pone.0279058)
Supplement: S1 Appendix — (DOCX) [file pone.0279058.s002.docx]

# Appendix Variables and equations

Eq. (1):

| $\boldsymbol{y=\Lambda\eta}+\nu$ |
| --- |

Eq. (2):

$\boldsymbol{\eta}=\boldsymbol{\Gamma x}+\zeta$,
